# Care of the Insane1Presidential address at the 37th annual meeting of the Medical Association of Central New York, held at Rochester, N. Y., September 20, 1904.

**Published:** 1904-12

**Authors:** C. A. Van Der Beek

**Affiliations:** Rochester, N.Y.


### BUFFALO MEDICAL JOURNAL.

VOL. XLIV.—LX. DECEMBER, 1904. NO. 5

### ORIGINAL COMMUNICATIONS.

### Care of the Insane.[1]

BY C. A. VAN DER BEEK, M. D., Rochester, N. Y.

PERMIT me at this time to thank you for the honor you have conferred upon me, by electing me your presiding officer for this, the thirty-seventh annual meeting of the Medical Association of Central New York.

This society had its inception in the thought and wisdom of such men as Dr. Didama, of Syracuse, Drs. Dean, C. E. Rider, Hovey, and the late Dr. E. M. Moore, of Rochester, and others from the counties of Cayuga, Ontario, Onondaga, Seneca, and Wayne.

The present membership of nearly 300, composed of representative physicians and surgeons of seventeen counties of Central and Western New York, and the inviting programs of scientific papers offered you from year to year, are a fitting tribute to that wisdom and foresight of our founders, whose desire it was to stimulate the study of the science and art of medicine.

The growth of the association has been a healthy one. The publication of its transactions, under the efficient management of Dr. William C. Krauss, now in their eleventh year, has been commended by many. The social gathering in the evening is a fitting terminal to the day's close application, and is of advantage to the association by promoting a better acquaintance among the physicians residing in this district.

The inviting program we are able to lay before you today came in response to the cards mailed you early in September, and

1. Presidential address at the 37th annual meeting of the Medical Association of Central New York, held at Rochester, N. Y., September 20, 1904.

it will soon be my privilege to give way to those whose subjects are of more interest to the average physician than is that which I will now present.

There are today in the various hospitals for the insane in this state, nearly 25,000 insane patients, and many more of this class are going about almost without restraint. The majority of these are of the harmless class. Many, however, are dangerous to themselves or others. Every one of this vast number has, at some period, been in the care of a physician engaged in general practice. It is to the latter to whom appeal is first made for guidance in dealing with this distressing malady, and it is often not an easy matter to determine offhand what should be done. A public, almost devoid of knowledge of disease, and one which is not easily influenced, even by the dramatic quality of events, has, largely in response to the selfish instinct for self-preservation, given us a number of institutions, called state hospitals, which, in fact, are largely asylums, and have made them easy and convenient receptacles for all classes of the insane.

It cannot be denied that the condition of the poor, as well as the prognosis, is improved by removal to these places, but I am frank to say, I believe it is not so with the middle or wealthy classes. I believe more can be done for them in the home than in the large hospital, and that their chances of recovery will be increased by keeping them away from a state institution. Have we, indeed, the right to subject a nondangerous and recoverable case to the humiliation of forcible detention in a public institution and the later suspicion and fear which an ignorant public always holds for a recovered mental case? I believe we have not.

The typhoid fever case is never rushed to a hospital, unless circumstances at home make it imperative, or the convenience of the medical attendant is enhanced thereby, though there are many hospitals especially designed for that class. The hospital is not essential to the obstetrical or surgical case. Why then, should it be to this other great class of pathological states, grouped under the general head of mental troubles?

A few years ago the superintendent of a New Jersey insane hospital recommended that cases of puerperal insanity be kept at home until every available recourse but the asylum had been tried without success.

Meynert lectured to his classes in Vienna to the effect, that in every instance there is a disadvantage in sending curable cases to an asylum. Mandsley affirms that "a large proportion of the curable insane can be treated to the best advantage at home, or in small asylums or homes," and another alienist summed up his vast experience in the treatment of the insane by saying "that it cannot be shown that the introduction of insane hospitals has added anything to the curability of insanity."

In the light of these authoritative views, and the facts well known to all who have carefully observed the effect of asylum influences upon the recovered cases, are we acting as true physicians or as conservers of the individual or public good when, through a spirit of commercialism, engendered by fear of trouble or the lack of time necessary to devote to the case, we advise immediate commitment?

We, as physicians, should discard the old theory that the insane require nothing more than sleep, and that, at whatever cost, but instead, we should know that a pathological change exists in this as in other forms of illness, which should be sought for and remedied, so far as is possible.

When our medical schools provide for a reasonable study of mental disorders, and make the passing of a final examination upon that subject equal in all respects to that of surgery or obstetrics, necessary before graduation, the subject will become far less dry, and physicians will be much better equipped to recognise disturbances of this character in their incipiency.

How many of the laity, or how many of our own number in fact, see in that symptom complex called neurasthenia anything more than the name implies; yet Dana asserts that 50 per cent. of cases so diagnosticated are, in realty, incipient cases of melancholia. And if it be true, as Clouston states, that all melancholias think of suicide, 20 per cent. attempt it, and 5 per cent. succeed, could not many curable cases be saved by the timely warning of the physician? It was but recently that the loss of a valuable life in a general hospital was caused by not recognising this element of melancholia in a supposed fatigue case.

It is a fact known to alienists that mental disturbances are very insidious in their onset, sometimes requiring weeks or months for their development, and that the prognosis is proportionally more favorable the earlier treatment is begun. We know the same principle obtains in other diseases, and we are constantly agitating for their prevention or early treatment. Why, then, in this doubly unfortunate class do we minimise the early warnings of a nervous trouble? I am sure many of the cases which now become organic and incurable could be checked in their incipiency if we better understood the toxin element in their etiology.

It is astonishing how we overlook the fact that alcohol causes directly a number of the insane, variously estimated at from 10 to 20 per cent., and syphilis produces large numbers of paretics annually, with its mortality of 100 per cent., while casting our influence toward state protection from the other terrible scourges of yellow fever, variola, tuberculosis and many more; we seem to shield our eyes from the dramatic quality of these trio of terrible visitors and refuse to draw a lesson from our protege Japan, where the dissemination of the latter disease is largely curtailed by detention hospitals, to which all venereal cases must go until cured.

The subject of mental disorders is dry because it is so little taught, and so much less studied, that an interest is seldom awakened, yet untold good might accrue, and the treatment of recoverable cases in the home become a pleasure instead of a burden, by the same consciousness of responsibility in our relations to the insane that we feel in reference to other maladies.

In every city there should be at least one general hospital equipped with a ward or pavilion specially designed for mental and nervous cases, to which it would be considered no stigma to be taken, and where we could temporarily or longer, treat these cases exactly as we now do those of a general medical nature. Such a ward could be made of inestimable value to the public, and to ourselves. It would give the house staff an opportunity to acquire some of the rudiments of mental disorders by association with them. It would enable the nurses to obtain some clinical experience, without which they are usually of little service, no matter from what training school they may have received their diplomas, and it would have a beneficent influence in disabusing the public mind of the unpardonable ignorance, recently so aptly expressed by the superintendent of a large general hospital when she said to a visitor, "Your brother is not ill, he is only crazy."

Our relations to the insane are far more comprehensive than this. The public looks to us for guidance, and we should fully meet the obligation. Many of the recent cases are prone to harm themselves and it is obviously our duty to protect them from the possibilities of their own faulty concepts. A few, and only a few, are dangerous to the family or to the public, and we should be able to quickly detect the signs which make it essential to remove the patient from home, and this we can readily do if we will familiarise ourselves with Spitzka's classification of delusions and hallucinations. We would then find some have a complex logical organisation and are called systematised, while others are devoid of such an organisation, are not as plausibly based, as elaborately expressed, or as skilfully defended, and are classed as the unsystematised variety. The former, when fixed and permanent, are characteristic of paranoia, an incurable, and sooner or later, dangerous malady. The unsystematised, if creative, and with elements of exaltation will be found in mania and maniacal states, and if to any of these be added many or varied visual hallucinations, usually of disagreeable objects, we are quite likely to have encountered a troublesome case.

If the fancies are creative, but with a predominating element of depression, and in some cases with visual or auditory hallucinations or illusions of single or few objects related to the patient's self, a melancholia will likely be present, although this class is found in the depressive phases of some chronic forms of mental disturbance. They may be monotonous and confused with elements of either exaltation or depression, and will then indicate either a defective development or a terminal state; or they may be monotonous and confused with elements of exaltation and depression mingled, and will then usually show an acute confusional form or one of pubescence, each safe and proper cases for home treatment.

Our individual responsibilities are not slight when we assume the care of an insane patient. It is not enough for us to say to the nurse, "You must be careful!" We should explain how, and that carefulness, as exercised in a typhoid case, might be carelessness with the insane. We should explain what to look for, and what to expect from this or that fancy. We should show her the value of persistence, of patience and of tact, all far more essential in mental than in body disorders. She must know that her presence will often avert trouble, and occasionally save life. We should impress upon her and upon the family, the fact that a sane and an insane person differ but little in their conceptions, and therefore merit the same careful attention and consideration, and when we, as medical practitioners, better understand the truth of the somatic element in the etiology of insanity, we will be better equipped for its early recognition, fewer cases will be certified, home treatment will receive greater attention, and with results more gratifying in every way to both patient and ourselves than we now obtain.

44 Gibbs Street.

---

### FOR PRURITUS VULVÆ.

| | | |
|---|---|---|
| ℞ | Acid borici | ʒii. |
| | Acid carbol. liq. | ʒi. |
| | Morphin. muriat. | gr. i. |
| | Vaselin | ℥ii. |

M. et ft. unguent. Sig.—Apply several times daily.

